# Cholestyramine treatment in two dogs with presumptive bile acid diarrhoea: a case report

**DOI:** 10.1186/s40575-021-00099-x

**Published:** 2021-01-19

**Authors:** L. Toresson, J. M. Steiner, J. S. Suchodolski

**Affiliations:** 1 Department of Equine and Small Animal Medicine, Faculty of Veterinary Medicine, Helsinki University, Agnes Sjobergin katu 2, 00014 Helsinki, Finland; 2 Evidensia Specialist Animal Hospital, Bergavagen 3, 25466 Helsingborg, Sweden; 3 Gastrointestinal Laboratory, Department of Small Animal Clinical Sciences, College of Veterinary Medicine and Biomedical Sciences, Texas A&M University, 4474 TAMU, College Station, TX 77843-4474 USA

**Keywords:** Bile acids, Diarrhoea, Dog, Cholestyramine

## Abstract

**Background:**

In people, bile acid diarrhoea is a prevalent complication of Crohn’s disease and diarrhoea-associated irritable bowel syndrome. Affected patients typically respond to bile acid sequestrants, such as cholestyramine, but human gastroenterologists often fail to recognize bile acid diarrhoea. Consequently, bile acid diarrhoea is regarded as an underrecognized and undertreated condition in human medicine. Due to lack of diagnostic tools, clinical response to bile acid sequestrants is often used to confirm a diagnosis of bile acid diarrhoea in people.

Several recent studies have shown that bile acid dysmetabolism also occurs in dogs with chronic enteropathies. It has further been shown that dogs with chronic enteropathies have significantly decreased expression of a bile acid transport protein in the ileum compared to healthy dogs, which correlates with faecal bile acid dysmetabolism. Consequently, in spite of the lack of reports in the literature, bile acid diarrhoea is likely to exist in dogs as well.

**Case descriptions:**

Two dogs, an 8-year old Rottweiler and a 4.5-year old Siberian Husky were evaluated for chronic watery diarrhoea. Neither dog responded to dietary trials, probiotics, cyclosporine, faecal microbial transplantations or metronidazole. One of the dogs responded to high daily doses of corticosteroids, which were however associated with unacceptable side effects. The other dog was refractory to all standard treatment protocols, including cyclosporine and corticosteroids. Since none of the dogs responded satisfactorily to standard treatment or modulation of the intestinal microbiome, a suspicion of possible bile acid diarrhoea was raised. Treatment with cholestyramine, a bile acid sequestrant was initiated and resulted in marked improvement of faecal consistency, frequency of defecation and activity level in both dogs.

**Conclusion:**

This report presents two dogs with presumed bile acid diarrhoea that were successfully treated with cholestyramine. Therefore, bile acid diarrhoea should be considered as a possible diagnosis in dogs with treatment-refractory chronic diarrhoea.

**Supplementary Information:**

The online version contains supplementary material available at 10.1186/s40575-021-00099-x.

## Plain English summary

Bile acids are produced by the liver and excreted in the small intestine where they are required for proper digestion of fat. They get absorbed in the last segment of the small intestine. In people with Crohn’s disease or irritable bowel syndrome associated with diarrhoea, absorption mechanisms fail, and excessive amounts of bile acids reach the large intestine where they can cause what is known as bile acid diarrhoea (BAD). Cholestyramine, a drug that binds bile acids, is used for treatment of BAD in people. Recent research has shown that dogs with chronic small intestinal inflammation may also have difficulty absorbing bile acids and end up with excessive concentrations in the large intestine. We describe two dogs with chronic diarrhoea that responded well to cholestyramine treatment when all other treatments had failed. We believe that BAD should be considered as a diagnosis in dogs with chronic small intestinal inflammation and diarrhoea that are refractory to standard treatment, and that cholestyramine treatment may be useful in these situations.

## Background

Primary bile acids (BAs) are synthesized in the liver and secreted into the small intestine, where they participate in fat absorption. Approximately 95% of BAs are reabsorbed in the ileum and undergo enterohepatic circulation [1]. This process is mediated by the apical sodium-dependent BA transporter (ASBT). Unabsorbed BAs are transformed by 7α-dehydroxylating colonic bacteria (e.g., *Clostridium hiranonis* in dogs) to secondary BAs [2]. In people, various chronic gastrointestinal disorders are associated with increased amounts of BAs in the colon. This stimulates electrolyte and water secretion and increases mucosal permeability and colonic motility [3]. Bile acid dysmetabolism and BA diarrhoea (BAD) have been reported in 40% of patients with Crohn’s disease and 32% of patients with diarrhoea-predominant irritable bowel syndrome (IBS-D) [4]. Patients with BAD typically respond to BA sequestrants (e.g., cholestyramine). However, despite a high prevalence, BAD is considered an underdiagnosed and undertreated condition in gastroenterology [5].

Bile acid dysmetabolism has recently been reported in several studies in dogs with chronic enteropathy (CE) [6–9]. Furthermore, decreased expression of the ASBT in the ileum of dogs with CE has been documented [7]. However, the authors could not find any published reports describing the clinical course and treatment of BAD in dogs. Therefore, the aim of this report was to describe the clinical presentation and response to cholestyramine treatment in two dogs with chronic refractory diarrhoea.

## Case presentation

### Case 1

An 8-year old neutered male Rottweiler with a body weight (BW) of 52 kg and a body condition score (BCS) of 5/9 was presented for a severe flare-up of watery diarrhoea and anorexia. The dog was already under treatment as an outpatient for previously diagnosed CE at a referral animal hospital, and was now admitted for fluid therapy and supportive care at the same animal hospital. On admission, the dog was on maintenance treatment with budesonide (3 mg on 2 days out of 3, Entocort; Tillotts Pharma GmbH, Rheinfelden, Germany) and a single protein source diet. The dog had been clinically stable with normal faeces for 2.5 years. Diarrhoea appeared once when budesonide had been tapered to 3 mg every other day, but was absent on a maintenance dose of 3 mg budesonide 2 days out of 3. Biopsies from the gastrointestinal tract had been collected endoscopically 4 years previously and showed mild to moderate lymphocytic-plasmacytic inflammation. Laboratory and imaging data at the time of admission to the animal hospital are shown in Tables 1 and 2.

**Table 1:** Selected serum biochemistry, haematology and faecal analysis

Parameter	Reference interval	Case 1	Case 2
Total leukocyte count	6.2–11.4 × 10^9^/L	10.8^a^	8.03^a^
Haematocrit	37.3–61.9%	44.9	**62.7**
Total protein	60–75 g/L	62	**55**
Albumin	29–39 g/L	34	32
Cholesterol	4.1–7.3 mmol/L	7.1	4.9
Alanine aminotransferase	0.3–1.3 ukat/L	0.8	0.7
Alkaline phosphatase	0.1–1.7 ukat/L	**54**	**1.8**
Creatinine	65–105 μmol/l	69	101
Blood urea nitrogen	3.8–9.0 mmol/L	**2.9**	6.7
C-reactive protein	0–30 mg/L	**45**	< 10
Cobalamin	180–700 pmol/l	468	405
Folate	15–45 nmol/l	> 54	16
Specific canine pancreatic lipase	0–200 μg/L	67	51
Trypsin-like immunoreactivity	5.5–35 μg/L	22	18
**Faecal tests**			
Faecal sedimentation-flotation	Negative	Negative	Negative
IDEXX SNAP Giardia test	Negative	Negative	Negative
Dysbiosis index	< 2	−0.7	N/A
Clostridium hiranonis	5.1–7.1 Log DNA/g	5.1	N/A

Bold numbers – results outside reference interval. ^a^Normal white blood cell differential count, N/A not applicable

**Table 2:** Gastrointestinal ultrasound report

Case 1	Duodenum: mildly thickened muscularis layer, mildly increased wall thickness. Jejunum: mild amount of mucosal speckles present in the oral part, mildly thickened muscularis layer in the aboral part. Mesenterial lymph nodes: mild enlargement, otherwise normal appearance. The remaining gastrointestinal tract within normal limits
Case 2	Jejunum: prominent muscularis layer in some areas. The remaining gastrointestinal tract within normal limits

Prednisolone (Prednisolon; Pfizer, Sollentuna, Sweden) at a dose of 10 mg q 24 h was added to the maintenance dose of budesonide for 3 weeks, which brought the diarrhoea temporarily into remission but led to calcinosis cutis, prompting cessation of prednisolone treatment and tapering of budesonide to 3 mg q 48 h. This was associated with recurrence of diarrhoea. The dog continued to have diarrhoea as well as excessive flatulence and halitosis during the following 12 months. Numerous treatment protocols, including additional immunosuppressive treatments such as cyclosporine and mycophenolate mofetil, metronidazole and dietary changes were tried at different times during this period (Table 3). A faecal microbiota transplantation was given once as a rectal enema at a dose of 5 g of frozen donor stool per kg body weight as described by Chaitman and co-workers [10]. The stool was thawed and blended with 0.9% saline on the day of administration. The donor dog was clinically healthy, free of intestinal parasites, bacterial pathogens and extended beta-lactamase resistant *E.coli*.

**Table 3:** Medical and dietary interventions besides corticosteroids prior to cholestyramine treatment

**Immunosuppressant**^**a**^	Case 1	Case 2
Cyclosporine (Cyclance vet; Virbac, Kolding, Denmark)	5 mg/kg q 24 h	N/A
Cyclosporine (Modulis Vet; Ceva, Lund, Sweden)	N/A	5 mg/kg q 24 h
Mycophenolate (Myfenax; Teva, Haarlem, the Netherlands)	10 mg/kg q 12 h	N/A
**Antibiotics**		
Metronidazole (Flagyl; Sanofi, Stockholm, Sweden)	10 mg/kg q 24 h	15 mg/kg q 24 h
**Miscellaneous drugs**		
Loperamide (Imodium; McNeil, Solna, Sweden)	0.08 mg/kg q 12 h	0.1 mg/kg q 12 h
Olsalazine (Dipentum; Pharmanovia, Basildon, Great Britain)	10 mg/kg q 12 h	15 mg/kg q 12 h
**Pro-and prebiotics**		
Mixed pre- and probiotic (Pro-fibre; Protexin vet)	15 g q 12 h	15 g q 12 h
Single strain probiotic (Fortiflora; Purina)	N/A	1 sachet q 24 h
Multistrain probiotic (Sivomixx; Ormendes)	1 sachet q 24 h	1 sachet q 24 h
Psyllium (Vi-Siblin; Meda)	30 ml q 12 h	N/A
**Diets**		
KD hydrolyzed protein diet (RC Hypoallergenic soy and rice)	Y	Y
KD hydrolyzed protein diet (Purina HA soy and corn)	Y	Y
KD hydrolyzed protein diet (Hill’s Z/D chicken and rice)	N/A	Y
KD hydrolyzed protein diet (Specific CDD-HY salmon and rice)	N/A	Y
KD single protein (RC Sensitivity control duck and tapioca)	Y	N/A
KD single protein (Hill’s D/D salmon and rice)	Y	N/A
KD fibre rich (Hill’s Gastrointestinal Biome Digestive)	Y	N/A
**Faecal Microbial Transplantation as rectal enema**		
Treatments	1	3

^a^add-on immunosupressants were given one at a time, N/A not applicable, KD kibble diet, Y dietary trial performed, RC Royal Canin

The dog was refractory to all treatments and had liquid diarrhoea 4–5 times daily, including once at night, and a canine inflammatory bowel disease activity (CIBDAI) index of 7 (consistent with moderate IBD) [11]. The poor response to medical and dietary intervention raised the suspicion of BAD. Cholestyramine (Kolestyramin Alternova; Orifarm Generics, Odense, Denmark) treatment was started with 2 g q 24 h for a week. The owners immediately noticed that the faeces became much firmer during 12 h after cholestyramine administration, but watery diarrhoea reappeared later during the day. The dose was increased to 2 g q 12 h, which further improved the quality of the faeces, reduced the frequency of defecation to 2–3 times daily, and markedly reduced flatulence. After increasing the dose to 3 g q 12 h, the diarrhoea stopped, CIBDAI had decreased to 2 (clinically insignificant disease) and faecal scores had improved from 5/5 (liquid diarrhoea) to 3/5 (formed but soft faeces) [12]. Dose reduction to 2 g cholestyramine q 12 h led to return of unformed feces within 36 h. The cholestyramine dose was again successfully increased to 3 g q 12 h. At the time of writing, diarrhoea had not recurred for 11 months, excessive flatulence had disappeared, and vomiting had ceased. Furthermore, the BCS had increased to 7/9 and the dog owner described the dog as more playful than during the past several years.

### Case 2

A 4.5-year old male neutered Siberian Husky with a BW of 34 kg and a BCS of 6.5/9 was referred for FMT. The dog had a life-long history of partially food-responsive chronic diarrhoea that had been somewhat stable as long as the dog only ate a hydrolyzed soy protein diet (Purina HA) with a probiotic supplement (Purina Fortiflora). Even when keeping a strict diet and using the probiotic supplement, flare-ups of diarrhoea and hyporexia occurred every week and lasted for 1–3 days. Several other diets had been tried previously, all resulting in diarrhoea (Table 3). The condition had deteriorated during the last 4 months to persistent diarrhoea, lethargy, marked hyporexia and 15% loss of BW. The dog defecated up to 7 times daily with a faecal score of 3–5/5 and had a CIBDAI score of 13 (severe IBD) [12]. For financial reasons, endoscopy had never been performed. Co-morbidities included zinc-responsive dermatosis and chronic blepharitis. Treatment with elemental zinc improved the skin condition partially. Neither cyclosporine treatment for 3.5 months, nor prednisolone improved the gastrointestinal signs. Metronidazole had been prescribed several times, without improvement of faecal quality. Laboratory and imaging data at the time of referral are shown in Tables 1 and 2. On physical examination, the dog was quiet with marked blepharitis, periorbital dermatitis and moderate facial crusting of the mucocutaneous junctions. The abdomen was moderately tense and painful on palpation. Liquid faeces were detected upon rectal palpation. FMT was given as a rectal enema, using the same donor and protocol as previously described, which initially was associated with a remarkable improvement regarding faecal quality, appetite, activity level and periorbital dermatitis [10]. However, ten days later, the dog appeared to have abdominal pain and diarrhoea returned, followed by pica, hyporexia and lethargy. Four days later, the dog came back for a second FMT. The same donor and protocol were used for the procedure, but no improvement of the gastrointestinal signs was noted. Budesonide treatment, 3 mg q 24 h, was initiated but led to more severe diarrhoea and was stopped after a few days. Loperamide was then prescribed without any improvement. A third FMT was performed 14 days later, without clinical improvement. At this time, cholestyramine at 2 g q 24 h was prescribed. Faecal quality and activity level improved during the first 12 h after each cholestyramine dose, but deteriorated during the following 12 h until the next dose. The cholestyramine dose was increased to 2 g q 12 h, which led to resolution of diarrhoea, decreased frequency of defecation, improved appetite and a more active and playful behaviour. Five months later at follow-up, twice daily cholestyramine treatment was still effective against diarrhoea, hyporexia and lethargy, and the CIBDAI score had decreased to 3 (clinically insignificant disease). Although occasional flare-ups of hyporexia and diarrhoea still occurred, these episodes were much shorter and occurred less frequently than before. Furthermore, the flare-ups of zinc-responsive dermatosis and chronic blepharitis occurred significantly less frequently than before.

## Discussion

This report describes successful treatment of chronic refractory diarrhoea with a BA sequestrant in two dogs. Recent studies have reported faecal BA dysmetabolism and decreased expression of ASBT in the ileum of dogs with CE [6–9]. Dogs with CE had significantly lower amount of total secondary BAs, and increased percentage of primary BAs compared to healthy dogs [7–9]. Similar BA dysmetabolism has been shown in people with inflammatory bowel disease too [13]. In another recent study, the serum concentration of 7α-hydroxy-4- cholesten-3-one (C4), a serum biomarkers of hepatic BA synthesis, was compared between healthy dogs and dogs with chronic diarrhoea [14]. Three of 17 dogs with chronic diarrhoea had serum C4 concentrations significantly above the calculated upper limit of the reference interval. These dogs were all partly or fully refractory to conventional therapy. Based on these previous studies it is likely that bile acid diarrhoea is a disease entity in dogs too, but reports on the clinical course and treatment of BAD in dogs are lacking.

It is estimated that 5–27% of dogs with CE have non-responsive enteropathy (NRE), if studies including only dogs with protein-losing enteropathy are excluded [15–23]. If dogs with food responsive enteropathy were excluded, the odds ratio (OR) of becoming refractory to treatment was significantly higher in steroid-responsive dogs compared to food-responsive dogs [16]. Potentially, some dogs with NRE may have BAD.

Bile acid diarrhoea is prevalent in humans with Crohn’s disease and IBS-D. Recent recommendations state that BAD should be considered early in patients with chronic diarrhoea [24]. Still, BAD remains an underrecognized and undertreated condition [5, 24]. There are several pathogenetic mechanisms described that lead to BAD in humans with gastrointestinal disorders, the most common of which is malabsorption of BAs in patients with ileal disease, and dysbiosis, which is associated with a decreased ability to convert primary BAs to secondary BAs. *Clostridium hiranonis* is a bacterial species that plays a prominent role in converting primary BAs to secondary BAs [2]. Decreased abundance of *C. hiranonis* has been reported in dogs with CE [9, 25]. However, one of the dogs in this report had a normal dysbiosis index and faecal *C. hiranonis* abundance, leaving malabsorption the most likely reason for BAD.

Treatment with glucocorticoids in rodent models and healthy human volunteers is associated with increased expression of ASBT, which likely increases BA reabsorptive capacity in the ileum [26, 27]. This positive effect on BA transport might have affected one of the case dogs as well, since diarrhoea ceased with higher doses of corticosteroids. However, this may also have been due to the anti-inflammatory and immune-suppressive properties of corticosteroids.

Cholestyramine is a sequestrant with a high affinity for BAs. When binding to BAs, an insoluble complex is formed that is excreted in the feces. Cholestyramine is recommended for use in dogs at an oral dose of 0.5–2.0 g/dog q 12 h for reduction of idiopathic hypercholesterolaemia [28]. Furthermore, cholestyramine has successfully been used to treat cyanobacterial toxicosis in a dog at a dose of 172 mg/kg for 17 days [29]. Several drugs and toxins must be bound to BAs to undergo enterohepatic circulation. Therefore, the irreversible binding of BAs to cholestyramine inhibits systemic toxin absorption and increases faecal excretion. In healthy laboratory Beagle dogs, the elimination rate of the non-steroidal anti-inflammatory drug (NSAID) tenoxicam was drastically accelerated when multiple doses of oral cholestyramine were given following tenoxicam injection, in contrast to both placebo and charcoal administration [30]. In Plumb’s Veterinary Drugs, cholestyramine is recommended to treat cyanotoxin exposure, NSAID toxicosis and vincristine overdose [31].

One recent study reported that a cholestyramine dose of 0.7 mg/kg q 24 h administered for 14 days to 12 healthy Beagle dogs appeared to be clinically safe [32]. No side effects or weight loss were noted. The faecal dry matter content increased with cholestyramine treatment, but the number of bowel movements did not increase and faecal scores were still in the normal range. Macronutrient apparent total tract digestibility decreased after cholestyramine treatment, but remained in the normal range. It should be noted that the dose used in these studies was 7 times higher than the doses used in this case report (0.058 mg/kg q 12 h and 0.059 mg/kg q 12 h, respectively). However, the long term consequences of cholestyramine treatment in dogs need to be studied.

In people, first-line treatment of BAD is cholestyramine, but gastrointestinal side effects, such as constipation, bloating, nausea, flatulence, abdominal pain and worsening diarrhoea are common [24]. This could affect compliance and make it difficult to titrate dosages to clinical effect. Side effects were less prominent and compliance was better when using newer and more expensive BA sequestrants such as colesevelam or colestipol. Besides gastrointestinal side effects, an over 3-fold increase in alanine aminotransferase (ALT) was noted in 11/67 healthy volunteers [33]. This increase was considered benign, and was not documented in any of the case dogs at follow-up visits. A few cases of vitamin K malabsorption and spontaneous bleeding have been reported in people treated long term with cholestyramine [34–37]. No negative effect on vitamin K absorption was noted in healthy laboratory Beagle dogs treated with dicumarol and Vitamin K when cholestyramine was given at a dose of 200 mg/kg q 24 h [38]. When the cholestyramine dose was increased to 1.0 g/kg, vitamin K absorption was somewhat delayed, but normalized within 24 h. A massive dose of 3.0 g/kg q 24 h of cholestyramine was associated with decreased absorption of vitamin K if the vitamin and cholestyramine was given at the same time, but not if cholestyramine was given 17 h prior to vitamin K.

In people, 3 categories of tests are available to confirm the diagnosis of BAD [39]. The SeHCAT test measures loss of fecal BAs and is considered the gold standard test, but is only available in very few laboratories worldwide [4]. Alternately, serum levels of of bile acid synthesis, including 7α-hydroxy-4-cholesten-3-one (C4) or the ileal regulatory hormone, fibroblast growth factor 19 (FGF19), can be measured by high-performance liquid chromatography (HPLC). These tests have good specificity and negative predictive value for BA malabsorption in patients with IBS-D or functional diarrhoea, but a lower sensitivity for other types of BA malabsorption. The C4 test is regarded as a good screening test to rule out BA malabsorption, but both C4 and FGF 19 have diurnal variation, which can cause false positive results [3, 40]. Measurement of faecal BAs can be performed using HPLC, but requires a 48 h stool collection period, which is not popular among patients [39]. These diagnostic tests are not widely available. Hence, clinical response to cholestyramine is often used to diagnose BAD in people [3]. This approach can be problematic, as a failed empiric trial with cholestyramine, often due to side effects, does not exclude BAD as a diagnosis [40]. However, the lack of tests to diagnose BAD should not exclude patients with chronic diarrhoea from empiric treatment.

In dogs, analysis of faecal BAs has been described, but a reference interval from a larger population of healthy dogs is lacking [6–9]. Until tools to diagnose BAD in veterinary medicine are validated and available, we hypothesize that, as in people, empirical treatment with BA sequestrants can be tried in dogs with chronic refractory diarrhoea. This treatment saved the two case dogs from euthanasia.

## Conclusion

This case report describes successful treatment of two dogs with chronic, long-lasting refractory diarrhoea with cholestyramine. Clinical response to cholestyramine is often used to diagnose BAD in people. Based on response to treatment of the two dogs in this study, we presume that the dogs in this report had BAD. Cholestyramine might serve as an alternative treatment option for dogs with intractable diarrhoea that do not respond to standard treatment protocols.

## Supplementary Information

**Additional file 1.**

## Data Availability

Data sharing is not applicable to this article as no datasets were generated or analysed during the current study, but details from the clinical records are available from the corresponding author on reasonable request.

## Abbreviations

ALT: Alanine aminotransferase
ASBT: Apical sodium-dependent bile acid transporter
BA: Bile acid
BAD: Bile acid diarrhoea
BCS: Body condition score
BW: Body weight
C4: 7α-hydroxy-4-cholesten-3-one
CIBDAI: Canine inflammatory bowel disease activity index
CE: Chronic enteropathy
FGF19: Fibroblast growth factor 19
FMT: Faecal microbiota transplantation
HPLC: High-performance liquid chromatography
IBD: Inflammatory bowel disease
IBS-D: Diarrhoea predominant irritable bowel syndrome
NRE: Non-responsive enteropathy
NSAID: Non-steroidal anti-inflammatory drug
q hrs: Every hours
SeHCAT: ^75^selenium homotaurocholic acid test
