# Correction to: Cholestyramine treatment in two dogs with presumptive bile acid diarrhoea: a case report

**DOI:** 10.1186/s40575-021-00102-5

**Published:** 2021-05-20

**Authors:** L. Toresson, J. M. Steiner, J. S. Suchodolski

**Affiliations:** 1 Department of Equine and Small Animal Medicine, Faculty of Veterinary Medicine, Helsinki University, Agnes Sjobergin katu 2, 00014 Helsinki, Finland; 2 Evidensia Specialist Animal Hospital, Bergavagen 3, 25466 Helsingborg, Sweden; 3 Gastrointestinal Laboratory, Department of Small Animal Clinical Sciences, College of Veterinary Medicine and Biomedical Sciences, Texas A&M University, 4474 TAMU, College Station, TX 77843-4474 USA

**Correction to: Canine Med Genet 8, 1 (2021)**

**https://doi.org/10.1186/s40575-021-00099-x**

Following publication of the original article [1], the authors identified an error that mg/kg should be g/kg in the Discussion section.

The correct sentences should read:

One recent study reported that a cholestyramine dose of 0.7 g/kg q 24 h administered for 14 days to 12 healthy Beagle dogs appeared to be clinically safe [32]. No side effects or weight loss were noted. The faecal dry matter content increased with cholestyramine treatment, but the number of bowel movements did not increase and faecal scores were still in the normal range. Macro- nutrient apparent total tract digestibility decreased after cholestyramine treatment, but remained in the normal range. It should be noted that the dose used in these studies was 7 times higher than the doses used in this case report (0.058 g/kg q 12 h and 0.059 g/kg q 12 h, respectively).

The original article [1] has been corrected.
