# Supplementary material for: Cholestyramine treatment in two dogs with presumptive bile acid diarrhoea: a case report
Source: Canine Med Genet. 2021 Jan 19;8:1. doi: 10.1186/s40575-021-00099-x (PMC7814458; doi:10.1186/s40575-021-00099-x)
Supplement: Supplementary file 1 — Additional file 1. [file 40575_2021_99_MOESM1_ESM.docx]

**Table 1.** The associations of NNMT expression with OS in GSE9891 dataset

| Variables | Number of patients | Univariate analysis | |  | Multivariate analysis | |
| --- | --- | --- | --- | --- | --- | --- |
|  |  | HR (95%CI) | P value |  | HR (95%CI) | P value |
| Age | 242 | 1.029 (1.008-1.051) | 0.006 |  | 1.031 (1.009-1.053) | 0.005 |
| Stage |  |  | 0.002 |  |  | 0.012 |
| Early | 37 | 1 |  |  | 1 |  |
| Late | 205 | 6.315 (1.997-19.963) |  |  | 4.444 (1.379-14.322) |  |
| Grade |  |  | 0.142 |  |  | / |
| Low | 99 | 1 |  |  | / |  |
| High | 143 | 1.365 (0.901-2.068) |  |  | / |  |
| Debulking |  |  | 0.005 |  |  | 0.054 |
| Optimal | 158 | 1 |  |  | 1 |  |
| Suboptimal | 84 | 1.770(1.190-2.633) |  |  | 1.494 (0.993-2.249) |  |
| NNMT expression |  |  | <0.001 |  |  | 0.002 |
| Low | 176 | 1 |  |  | 1 |  |
| High | 66 | 2.278 (1.513-3.430) |  |  | 1.914 (1.265-2.894) |  |
